# Electrical Impedance Spectroscopy-Based Defect Sensing Technique in Estimating Cracks

**DOI:** 10.3390/s150510909

**Published:** 2015-05-08

**Authors:** Tingting Zhang, Liangdong Zhou, Habib Ammari, Jin Keun Seo

**Affiliations:** 1 Department of Computational Science and Engineering, Yonsei University, Seoul 120-749, Korea; E-Mails: zttouc@hotmail.com (T.Z.); zhould1990@hotmail.com (L.Z.); 2 Department of Mathematics and Applications, Ecole Normale Supérieure, 45 Rue d'Ulm, 75005 Paris, France; E-Mail: habib.ammari@ens.fr

**Keywords:** defect sensing, impedance spectroscopy, inverse problem, concrete cracks

## Abstract

A defect sensing method based on electrical impedance spectroscopy is proposed to image cracks and reinforcing bars in concrete structures. The method utilizes the frequency-dependent behavior of thin insulating cracks: low-frequency electrical currents are blocked by insulating cracks, whereas high-frequency currents can pass through thin cracks to probe the conducting bars. From various frequency-dependent electrical impedance tomography (EIT) images, we can show its advantage in terms of detecting both thin cracks with their thickness and bars. We perform numerical simulations and phantom experiments to support the feasibility of the proposed method.

## 1. Introduction

As a number of concrete structures currently in service reach the end of their expected serviceable life, nondestructive testing (NDT) methods to evaluate their durability, and, thus, to ensure their structural integrity, have received gradually increasing attention. Concrete often degrades by the corrosion of the embedded reinforcing bars, which can lead to internal stress and, thus, to structurally-disruptive cracks [1]. Various NDT techniques are currently used to monitor the reliability and condition of reinforced concrete structures without causing damage. They include impact-echo, half-cell potential, electrical resistivity testing, ground-penetrating radar, ultrasonic testing, infrared thermographic techniques and related tomographic imaging techniques [1–16]. Each technique has its intrinsic limitations in terms of the reliability of defect detection, and the conventional techniques often depend on the subjective judgment of the inspectors. The limitations of existing methods have led to the search for more advanced visual inspection methods to detect invisible flaws and defects on the surface of concrete structures.

Electric methods, such as electrical resistivity tomography (ERT) and electrical capacitance tomography (ECT), have been used to image cracks and steel reinforcing bars, which show clear electrical contrast from the background concrete [15–20]. These electric methods can be used to complement acoustic methods by assessing different characteristics. They operate at low cost over long time periods. ERT and ECT employ multiple current sources to inject currents, and boundary voltages are then measured using voltmeters connected to multiple surface electrodes on the boundary of the imaging subject. These methods use the relationship between the applied current and the measured boundary voltage to invert the image of cracks and reinforcing bars. The methods suffer from a low defect location accuracy due to the ill-posedness of the corresponding inverse problem. Moreover, most of the previous electric methods with a single frequency may have difficulty identifying both cracks and reinforcing bars simultaneously. In addition to the above electrical techniques, frequency selective circuits (FSC) and electrical impedance spectroscopy (EIS) using multiple frequencies have been applied to detect cracks and damages in concrete materials [6,8–11].

In this work, we present an impedance-spectroscopy-based NDT method for imaging both cracks and bars using multi-frequency electrical impedance tomography (mfEIT) at various frequencies ranging between 10 Hz and 1 MHz. The method is based on a mathematical understanding of the frequency-dependent behaviors of thin insulating cracks and reinforcing bars: low-frequency electrical currents are blocked by insulating cracks, whereas high-frequency currents can pass through thin cracks to probe the conducting bars. We make use of an elliptic interface problem to explain how high-frequency current can penetrate the thin cracks in terms of their thickness. The proposed impedance spectroscopy-based NDT method increases the amount of information of cracks by providing visual assessment of the condition of a concrete structure at various frequencies. The numerical simulations use a conventional 16-channel EIT system, with the electrical current applied between two adjacent electrodes at different frequencies. The cracks are modeled as thin homogeneous insulating films, while the reinforcing bars are considered as perfectly conducting materials. The boundary voltage data are then measured between two adjacent electrodes attached on the surface. Multi-frequency EIT reconstruction at various frequencies allows the detection of both the cracks and the reinforcing bars within the concrete structures. Numerical experiments and phantom experiment are presented here to illustrate the main findings.

## 2. Methods

**Figure 1:**
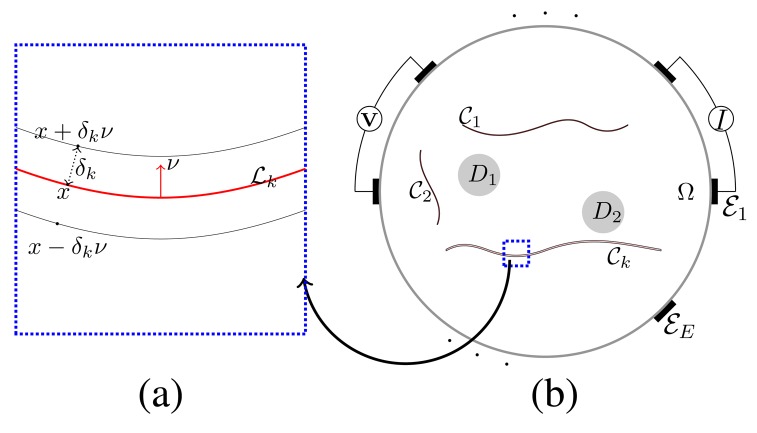
Schematic illustration of a multi-frequency electrical impedance tomography (mfEIT) model with highly conductive reinforcing bars *D*_1_, *D*_2_ and cracks 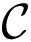
_1_, 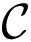
_2_, 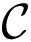
*_k_*: (**a**) crack 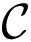
*_k_* is a tubular neighborhood of curve 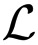
*_k_*; (**b**) configuration of mfEIT model.

Let Ω denote the two-dimensional cross-section of an imaging object, including reinforcing bars and concrete cracks. Denote by $C=\cup_{j=1}^{N}C_{j}$ the region occupying the collection of cracks, and let $D=\cup_{j=1}^{M}D_{j}$ be the region occupying the collection of bars. Figure 1 shows the cross-sectional domain, including two reinforcing bars *D*_1_, *D*_2_ and cracks 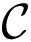
_1_, 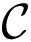
_2_, 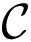
*_k_*. In the mfEIT system, we attached electrodes 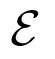
*_j_* for *j* = 1,⋯, *E* on the boundary *∂*Ω and inject a sinusoidal current with an angular frequency *ω* (0 ≤ *ω*/2*π* ≤ 10^6^) through a chosen pair of electrodes.

We consider the two-dimensional model with the axial symmetry assumption by transversally injecting current in the imaging slice. Then, the resulting time-harmonic complex potential *u^ω^* is dictated approximately by:

(1) $$\{\begin{array}{cc} \nabla \cdot \left(\gamma^{\omega }\nabla u^{\omega }\right)=0 & \text{in}\;\Omega \\ \gamma^{\omega }\frac{\partial u^{\omega }}{\partial \nu }=g & \text{on}\;\partial \Omega \end{array}$$

where *γ*^ω^ is the admittivity distribution, *ν* is the outward unit normal vector and *g* is the magnitude of the current density on *∂*Ω due to the injection current [21]. On current injection electrodes 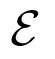
_1_ and 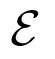
_2_, we have *∫*_
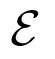
_1__ *g ds* = *I* = − *∫*_
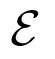
_2__ *g ds*, where *ds* is the surface element. The Neumann data *g* are zero on the regions of boundary not contacting with current injection electrodes. We measure the resulting boundary potentials at the remaining adjacent pairs of electrodes; $V_{\omega }^{1,3},\cdots ,V_{\omega }^{1,E-1}$, where $V_{\omega }^{1,k}$ denotes the voltage between 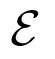
*_k_* and 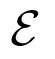
*_k_*_+1_. Subsequently, the second injection current is applied using the next pair 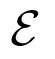
_2_ and 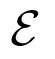
_3_, and we obtain the resulting voltage data $\left(V_{\omega }^{2,4},\cdots ,V_{\omega }^{2,E}\right)$. Performing this process for all pairs of electrodes creates current-voltage data having *E*(*E* − 3) values:

$${\text{F}}_{\omega }=\left(V_{\omega }^{1,3},\cdots ,V_{\omega }^{1,E-1};V_{\omega }^{2,4},\cdots ,\cdots \right)$$

In the Appendix, we provide a detailed explanation of the current-voltage data **F***_ω_*. The inverse problem is to identify the structure of cracks and reinforcing bars from the multiple frequency dataset **F**_*ω*_1__, …, **F***_ω_K__*

**Figure 2:**
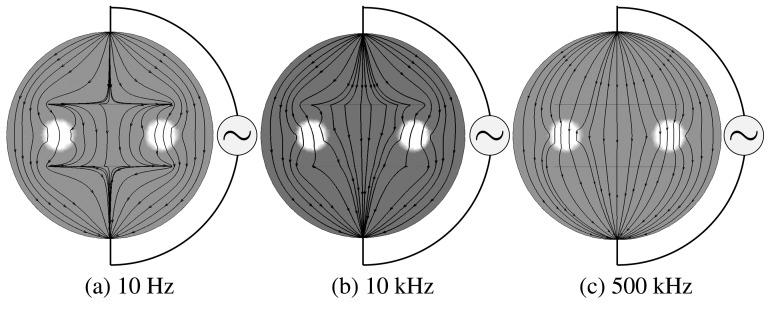
Changes of electrical current flows near cracks with frequencies: (**a**) 10 Hz, (**b**) 10 kHz and (**c**) 500 kHz.

Figure 2 shows the frequency-dependent behavior of the electrical current flows near cracks. The proposed method takes advantage of a noticeable change of the reflection of the complex potential associated with the frequency *ω* across the cracks. However, due to complicated coupling of the complex solution *u^ω^* of the PDE Equation (1) between its real and imaginary part, it is very difficult to analyze how locations and shapes of cracks, as well as their thickness are linked to the frequency-dependent behavior of the complex potential. In the presence of very thin insulating cracks, numerical approaches using finite element methods require a huge amount of computational effort. It would be desirable to develop a simplified model for better intuition and analysis to look at reflection associated with the abrupt conductivity changes across the cracks.

In order to emphasize the abrupt changes in the admittivity distribution *γ^ω^* across the boundaries of reinforcing bars and concrete cracks, we denote:

(2) $$\gamma^{\omega }=\{\begin{array}{cc} \gamma_{c}^{\omega }=\sigma_{c}+i\omega \epsilon_{c} & \text{in}\;C\\ \gamma_{d}^{\omega }=\sigma_{d}+i\omega \epsilon_{d} & \text{in}\;D\\ \gamma_{b}^{\omega }=\sigma_{b}+i\omega \epsilon_{b} & \text{otherwise} \end{array}$$

Assuming that the cracks are highly insulating and the reinforcing bars are highly conducting, we consider the following two extreme contrast cases: *σ_c_/σ_b_* ≈ 0 and *σ_d_/σ_b_* ≈ ∞.

Now, we are ready to present an insightful model explaining the frequency-dependent behavior of *u^ω^* around cracks. For simplicity, we assume that each crack 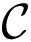
*_k_* is a tubular neighborhood of a smooth open curve 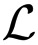
*_k_* with a uniform thickness *δ_k_*, as shown in Figure 1a. We consider that the following model whose solution *ũ^ω^* can be viewed as a reasonably good approximation of the potential *u^ω^* in Equation (1):

(3) $$\nabla \cdot \left(\gamma_{b}^{\omega }+\left(\gamma_{d}^{\omega }-\gamma_{b}^{\omega }\right)\chi_{D}\right)\nabla {\tilde{u}}^{\omega }=0\;\text{in}\;\Omega \backslash \cup_{k=1}^{N}L_{k}$$

(4) $$\left[{\tilde{u}}^{\omega }\right]-\beta_{k}\left(\omega \right)\frac{\partial {\tilde{u}}^{\omega }}{\partial \nu }=0\;\text{on}\;{\text{L}}_{k}\left(k=1,\cdots ,N\right)$$

(5) $$\left[\frac{\partial }{\partial \nu }{\tilde{u}}^{\omega }\right]=0\;\text{on}\;{\text{L}}_{k}\left(k=1,\cdots ,N\right)$$

(6) $$\gamma_{b}^{\omega }\frac{\partial {\tilde{u}}^{\omega }}{\partial \nu }=g\;\text{on}\;\partial \Omega$$

where

(7) $$\beta_{k}\left(\omega \right)=2\delta_{k}\frac{\sigma_{b}+i\omega \epsilon_{b}}{\sigma_{c}+i\omega \epsilon_{c}}$$

and the bracket [*ϕ*] indicates a jump across the interface 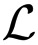
*_k_*; for *x* ∈ 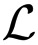
*_k_*,

(8) $$\left[\phi \right]\left(x\right):=\lim_{s\to 0+}\left\{\phi \left(x+s\nu_{x}\right)-\phi \left(x-s\nu_{x}\right)\right\}$$

Here, *χ_D_* denotes the characteristic function of *D* that takes one in *D* and zero otherwise. Various numerical simulations show that *u^ω^* ≈ *ũ^ω^* near the boundary Ω.

This model, Equation (3) to Equation (6), allows us to understand the relationship between the frequency-dependent behavior of the current-voltage data and the character of cracks, including their thickness. The current-voltage data are mainly affected by the outermost cracks when the frequency is low, whereas the data mainly depend on the reinforcing bars when the frequency is high. For this reason, we can detect the outermost cracks at low frequency. As frequency increases, the reinforcing bars become gradually visible, whereas cracks fade out (thicker crack fades out at higher frequency than thinner crack). Hence, a multi-frequency EIT system allows one to probe these frequency dependent behavior.

### Remark 1

*The interface condition of Equation (4) to Equation (5) explains the frequency-dependent behavior of the complex potential. To see this behavior clearly, let us assume:*

(9) $$\sigma_{c}/\sigma_{b}\leq 10^{-6},10^{-3}\leq \epsilon_{c}/\epsilon_{b}\leq 1,\epsilon_{b}/\sigma_{c}\leq 10^{-2}$$

*According to Equation (7), the parameter β_k_(ω) as a function of ω is approximated as:*

(10) $$\begin{array}{c} \beta_{k}(\omega )\approx 2\delta_{k}\sigma_{b}/\sigma_{c}\;if\;\omega /2\pi \leq 1\;\text{kHz},\\ \beta_{k}(\omega )\approx 2\delta_{k}\left(\epsilon_{b}/\epsilon_{c}-i\sigma_{b}/\omega \epsilon_{c}\right)\;if\;\omega /2\pi \geq 100\;\text{kHz}. \end{array}$$

*For a fixed crack thickness having δ_k_σ_b_/σ_c_* ≫ 1, *the magnitude of β_k_(ω) at low frequency is very large. Hence, at low frequencies below 1 kHz, the jump condition in Equation (4) can be regarded as the homogeneous Neumann boundary condition:*

(11) $$\frac{\partial {\tilde{u}}^{\omega }}{\partial \nu }=0\;on\;L_{k}\left(k=1,\cdots ,N\right).$$

*As frequency grows, the magnitude of β_k_(ω) decreases. As a result, potential jump is decreased across the interface 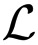
_k_* (*k* = 1,⋯, *N*). *Furthermore, for a fixed current frequency ω, β_k_(ω) is directly proportional to crack thickness*.

Now, we provide numerical validation for the approximation of *u^ω^* ≈ *ũ^ω^* near the boundary Ω. Recalling that 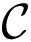
*_k_* = {*y* : *y* = *x* + *sν_x_*, − *δ_k_* < *s* < *δ_k_, x* ∈ 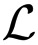
*_k_*}, the jump of *u^ω^* along two sidewalls of crack 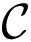
*_k_* is given by:

$$〚u^{\omega }〛\left(x\right):=\underset{s\to 0^{+}}{\lim}u^{\omega }\left(x+\left(\delta_{k}+s\right)\nu_{x}\right)-u^{\omega }\left(x+\left(\delta_{k}+s\right)\nu_{x}\right)$$

for *x* ∈ 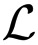
*_k_*. We define $〚\frac{\partial u^{\omega }}{\partial \nu }〛$ in the same way. Then, it follows from the transmission condition across two sidewalls of cracks 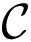
*_k_* that the following jump conditions can be inferred:

(12) $$\begin{array}{r} {〚u^{\omega }〛\left(x\right)-\beta_{k}\left(\omega \right)\frac{\partial u^{\omega }}{\partial \nu }\left(x-\delta_{k}\nu_{x}\right)|}_{+}\approx 0\\ \text{on}\;{\text{L}}_{k}\left(k=1,\cdots ,N\right) \end{array}$$

(13) $$〚\frac{\partial u^{\omega }}{\partial \nu }〛\left(x\right)\approx 0\;\text{on}\;{\text{L}}_{k}\left(k=1,\cdots ,N\right)$$

where:

$${\frac{\partial u^{\omega }}{\partial \nu }\left(x-\delta_{k}\nu_{x}\right)|}_{+}=\underset{s\to 0^{+}}{\lim}\frac{\partial u^{\omega }}{\partial \nu }\left(x-\left(\delta_{k}+s\right)\nu_{x}\right)$$

**Figure 3:**
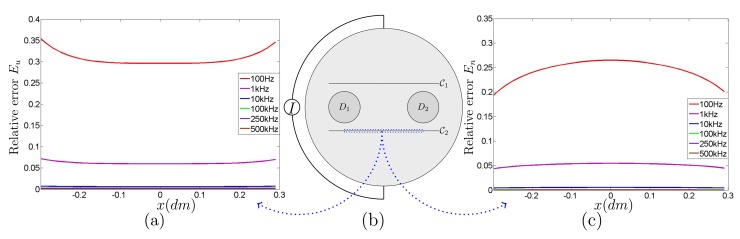
Numerical validation of interface jump conditions of Equations (4) and (5): (**a**) relative error $E_{u}=\left|{〚u^{\omega }〛\left(x\right)-\beta_{k}\left(\omega \right)\frac{\partial u^{\omega }}{\partial \nu }\left(x-\delta_{k}\nu_{x}\right)|}_{+}\right|/\left|〚u^{\omega }〛\left(x\right)\right|$ for *x* ∈ 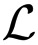
_2_ on dotted area of (**b**) at the frequencies 100 Hz, 1 kHz, 10 kHz, 100 kHz, 250 kHz and 500 kHz; (**c**) Relative error $E_{n}=\left|〚\frac{\partial u^{\omega }}{\partial \nu }〛\left(x\right)\right|/\left|〚\frac{\partial u^{\omega }}{\partial \nu }〛\left(x\right)\right|$ for *x* ∈ 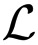
_2_ on dotted area of (**b**) at the frequencies 100 Hz, 1 kHz, 10 kHz, 100 kHz, 250 kHz and 500 kHz.

Figure 3 provides the numerical validation of interface conditions in Equations (12) and (13) at the frequencies 100 Hz, 1 kHz, 10 kHz, 100 kHz, 250 kHz and 500 kHz. The potential *u^ω^* is computed by FEM to obtain $〚u^{\omega }〛\left(x\right)-{\beta_{k}\left(\omega \right)\frac{\partial u^{\omega }}{\partial \nu }\left(x-\delta_{k}\nu_{x}\right)|}_{+}$ and $〚\frac{\partial u^{\omega }}{\partial \nu }〛\left(x\right)$ for *x* in the dotted area of 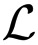
_k_. Figure 3a indicates the relative error $|〚u^{\omega }〛\left(x\right)-{\beta_{k}\left(\omega \right)\frac{\partial u^{\omega }}{\partial \nu }\left(x-\delta_{k}\nu_{x}\right)|}_{+}|/\left|〚u^{\omega }〛\left(x\right)\right|$ at various frequencies of 100 Hz, 1 kHz, 10 kHz,100 kHz, 250 kHz and 500 kHz. Figure 3c shows the relative error $〚\frac{\partial u^{\omega }}{\partial \nu }\left(x\right)〛$ with respect to $\left|\frac{\partial u^{\omega }}{\partial \nu }\left(x\right)\right|$ over the dotted area of 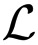
*_k_*. This numerical simulation shows that the relative errors are less than 10^−1^. Additively, when frequency is above 10 kHz, the relative errors are less than 10^−3^. Hence, *ũ^ω^* is reasonably close to *u^ω^* in a region away from the cracks. (At low frequencies below about 1 kHz, |*β_k_*(*ω*)| is very large, so that the jump condition in Equation (4) can be regarded as $\frac{\partial {\tilde{u}}^{\omega }}{\partial \nu }\approx 0$ on 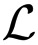
_2_. According to the transmission condition of *u^ω^* and the assumption of *σ_c_*/*σ_b_* ≈ 0, we have $\frac{\partial {\tilde{u}}^{\omega }}{\partial \nu }=0$, and therefore, *u^ω^* ≈ *ũ^ω^* in a region away from the cracks.)

In very special cases, we compute the interface conditions of Equations (4) and (5) explicitly. The following two remarks consider two special cases; the one-dimensional model (Remark 2) and the two-dimensional circular cracks (Remark 3).

### Remark 2

*Consider the simplest one-dimensional crack model by regarding the interval* (*x*_0_ − *δ, x*_0_ + *δ*) *as a crack in the interval* (0, 1). *If u^ω^ satisfies:*

$$\frac{d}{dx}\left(\left(\gamma_{b}^{\omega }+\left(\gamma_{c}^{\omega }-\gamma_{b}^{\omega }\right)\chi_{\left(x_{0}-\delta ,x_{0}+\delta \right)}\right)\frac{d}{dx}u^{\omega }\right)=0\;in\;\left(0,1\right)$$

*then a direct computation yields the following jump conditions along cracks:*

$$\begin{array}{c} 〚u^{\omega }〛\left(x_{0}\right)={\beta_{\delta }\left(\omega \right)\frac{du^{\omega }}{dx}\left(x_{0}+\delta \right)|}_{+},\\ 〚\frac{du^{\omega }}{dx}〛\left(x_{0}\right)=\frac{du^{\omega }}{dx}\left(x_{0}+\delta \right)-\frac{du^{\omega }}{dx}\left(x_{0}-\delta \right)=0. \end{array}$$

**Figure 4:**
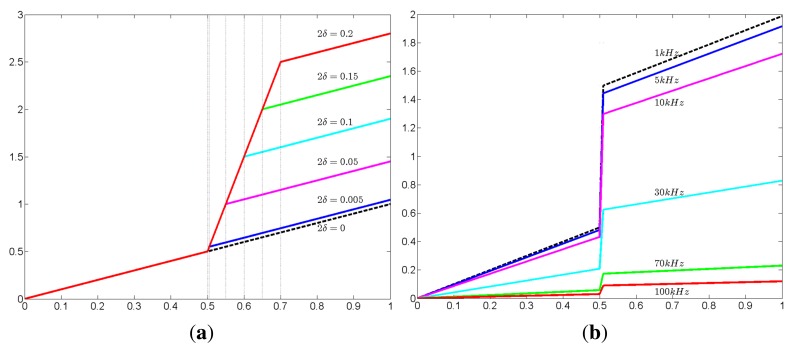
The interface jump of potential 〚*u^ω^*〛 across interval (*x*_0_ − *δ, x*_0_ + *δ*) at various thicknesses and frequencies; the real part of the potential *u^ω^* (y-axis) in Example 2 with respect to (**a**) six different thicknesses 2*δ* = 0.2, 0.15, 0.1, 0.05, 0.005, 0 and (**b**) six different current frequencies *ω*/2π = 1 kHz, 5 kHz, 10 kHz, 30 kHz, 70 kHz, 100 kHz. Here, the boundary condition is *u^ω^*(0) = 0, $\gamma_{b}^{\omega }\frac{d}{dx}u^{\omega }\left(0\right)=1$ and $\gamma_{b}^{\omega }\frac{d}{dx}u^{\omega }\left(1\right)=-1$.

*Here*, $\beta_{\delta }\left(\omega \right)=2\delta \gamma_{b}^{\omega }/\gamma_{c}^{\omega }$. *Figure 4 shows the interface jumps of potential*〚*u^ω^*〛 *across interval*(*x*_0_ − *δ, x*_0_ + *δ*) *at different frequencies and different thicknesses*.

### Remark 3

*Assume that the two-dimensional domain* Ω *contains the circular crack 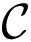 given by 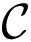
* = {*x* : *r*_0_ − *δ < |x| < r*_0_ + *δ*}. *Assume r*_0_ + *δ < r*_1_ *and*{*x* : *|x| < r*_1_} ⊂ Ω. *Consider the potential u^ω^ satisfying:*

$$\nabla \cdot \left(\left(\gamma_{b}^{\omega }+\left(\gamma_{c}^{\omega }-\gamma_{b}^{\omega }\right)\chi_{C}\right)\nabla u^{\omega }\right)=0\;in\;\Omega .$$

*Using the transmission condition across ∂ 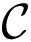 and separation of variables, we can express u^ω^ as the following form:*

$$u^{\omega }\left(r,\theta \right)=\{\begin{array}{cc} a_{0}^{1}+\Psi \left(r,\theta \right), & if0\;\leq r\leq r_{0}-\delta ,\\ a_{0}^{2}+\Psi \left(r,\theta \right)\gamma_{b}^{\omega }/\gamma_{c}^{\omega }, & if\;r_{0}-\delta \leq r<r_{0}+\delta ,\\ a_{0}^{3}+\Psi \left(r,\theta \right) & if\;r_{0}+\delta \leq r<r_{1}, \end{array}$$

*where* $\Psi \left(r,\theta \right)=\sum_{n=1}^{\infty }\left(a_{n}r^{n}\cos n\theta +b_{n}r^{n}\sin n\theta \right)$ *and* $a_{0}^{3}-a_{0}^{1}=\left(\gamma_{b}^{\omega }/\gamma_{c}^{\omega }-1\right)\left(\Psi \left(r_{0}+\delta ,\theta \right)-\Psi \left(r_{0}-\delta ,\theta \right)\right)$*. Then, we have the following jump relations across the ring interface:*

$$〚\frac{\partial u^{\omega }}{\partial \nu }〛\left(r_{0},\theta \right)=O\left(\delta \right)$$

*and*

$$\begin{array}{cc} 〚u^{\omega }〛\left(r_{0},\theta \right) & =\left(\Psi \left(r_{0}+\delta ,\theta \right)-\Psi \left(r_{0}-\delta ,\theta \right)\right)\gamma_{b}^{\omega }/\gamma_{c}^{\omega },\\ & =\beta_{\delta }\left(\omega \right)\frac{\partial u^{\omega }}{\partial \nu }{\left(r_{0}+\delta ,\theta \right)|}_{+}+O\left(\delta^{2}\right). \end{array}$$

## 3. Results

Numerical simulations and phantom experiments are carried out to verify the feasibility of the multi-frequency method of detecting cracks and reinforcing bars.

### 3.1. Numerical Simulations

**Figure 5:**
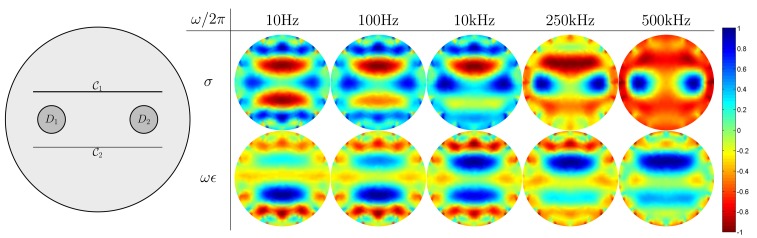
Reconstructed admittivity images using the multi-frequency EIT method. The second row is reconstructed images for normalized sigma *σ* (S/m), and the third row is reconstructed images for normalized *ωϵ* (S/m).

**Figure 6:**
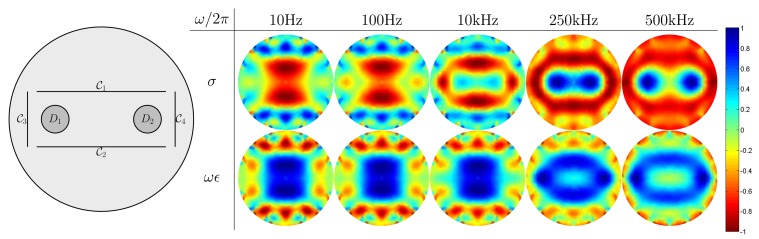
Reconstructed admittivity images using the multi-frequency EIT method. The second row is reconstructed images for normalized sigma *σ* (S/m), and the third row is reconstructed images for normalized *ωϵ* (S/m).

**Figure 7:**
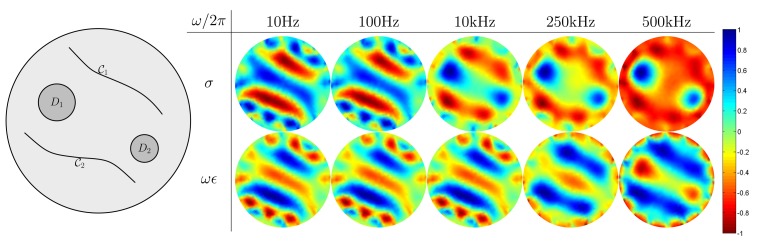
Reconstructed admittivity images using the mfEIT method using reference data from the homogeneous admittivity distribution. The second row is reconstructed images for normalized sigma *σ* (S/m), and the third row is reconstructed images for normalized *ωϵ* (S/m).

We make use of three different numerical simulation models on a disk Ω = { (*x, y*) : *x^2^+y*^2^ ≤ (0.1)^2^} with the radius of 0.1 m, as shown in Figures 5–7. Inside the disk, we placed cracks and bars. The complex admittivity distribution for each model is chosen as shown in Table 1.

In these numerical simulations, we used five different frequencies of 10 Hz, 100 Hz, 10 kHz, 250 kHz and 500 kHz for mfEIT image reconstructions. We use FEM to solve the forward problem (1) and generate simulated current-voltage data {**F**_*ω*_1__,**F**_*ω*_2__, **F**_*ω*_3__, **F**_*ω*_4__, **F**_*ω*_5__}. Using the standard mfEIT image reconstruction method [21,22], we provide images of spectroscopic conductivity distribution *σ* (S/m) and *ωϵ* (S/m) at various frequencies, and all reconstructed images are normalized ranging from −1 to 1.

Figure 5 shows the reconstructed images at the five frequencies in the case when there are two reinforcing bars *D*_1_ *=* { (*x, y*) : (*x*+0.05)^2^+y^2^ ≤ (0.015)^2^}, *D*_2_ = { (*x, y*) : (*x*−0.05)^2^+y^2^ ≤ (0.015)^2^} and two thin concrete cracks 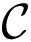
_1_ = {(*x, y*) : |*x*| < 0.07, |*y* + 0.03| < 5 × 10^−5^}, 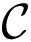
_2_ = {(*x, y*) : |*x*| < 0.07, |*y* + 0.03| < 2.5 × 10^−5^}. At low frequencies (10 Hz, 100 Hz), the injected electrical currents are detoured around the insulating cracks. Consequently, the current-voltage data are mainly influenced by the concrete cracks at low frequencies (10 Hz, 100 Hz), and the cracks are only visible in the reconstructed images. On the other hand, the injected currents at the high frequencies (250 kHz, 500 kHz) penetrate the cracks. Hence, the current-voltage data at the high frequencies are mainly influenced by the bars, so that the cracks are invisible. From the spectroscopic images, we could get the information of both cracks and bars, including the thickness of cracks.

In Figure 6, two reinforcing bars *D*_1_ = {(*x, y*) : (*x* + 0.05)^2^ + *y*^2^ ≤ (0.015)^2^}, *D*_2_ = { (*x, y*) : (*x*−0.05)^2^+*y*^2^ ≤ (0.015)^2^} are encircled by four concrete cracks; 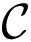
_1_ = {(*x, y*) : |*x*| < 0.07, |*y*−0.03| < 2.5 × 10^−5^}, 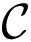
_2_ *=* {(*x, y*) : |*x*| < 0.07, |*y* + 0.03| < 2.5 × 10*^−^*^5^}, 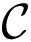
_3_ = {(*x, y*) : |*x* + 0.08| < 2.5 × 10^−5^, |*y*| < 0.03}, 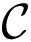
_4_ = {(*x, y*) : |*x*− 0.08| < 2.5 × 10^−5^, |*y*| < 0.03}.

The simulation results show that at a low frequency, the four outermost encircled cracks appear to be one object, whereas reinforcing bars are hidden by cracks, because the injected currents are blocked by the insulating cracks. As the frequency increases, cracks gradually disappear, whereas reinforcing bars begin to show up.

In Figure 7, there are two curved cracks with their thickness 5 × 10^−5^ *m* and two reinforcing bars *D*_1_ = { (*x, y*) : (*x* + 0.045)^2^ + (*y* − 0.02)^2^ < (0.02)^2^}, *D*_2_ = {(*x, y*) : (*x* − 0.05)^2^ + (*y* + 0.03)^2^ ≤ (0.015)^2^}. The simulation results show a similar behavior as in the previous cases. All of the numerical simulation results are consistent with observations in the previous section.

**Figure 8:**
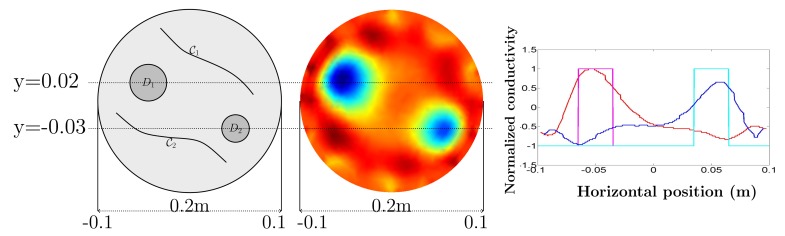
Profiles of a normalized conductivity image of reinforcing bars at the frequency 500 kHz along the x directions (*y* = 0.02 and *y* = −0.03), where the radius of Ω is 0.1 m; the magenta (cyan) line is for true conductivity values, and the red (blue) line is for the reconstructed conductivity values along *y* = 0.02 (*y* = −0.03).

Furthermore, we provide the profiles of a normalized conductivity distribution of reinforcing bars at the frequency 500 kHz along the x directions *y* = 0.02 and *y* = −0.03, as shown in Figure 8, where it is obvious that the highest intensity implies the center of reinforcing bars *D*_1_, *D*_2_ in the redirection.

### 3.2. Phantom Experiments

**Figure 9:**
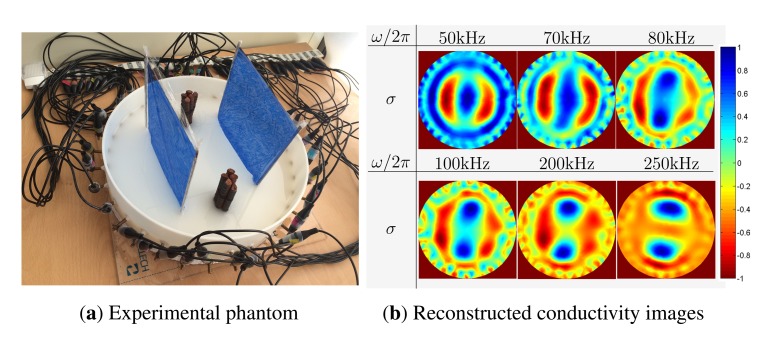
Phantom experiments. (**a**) Phantom with two reinforcing bars and two thin plastic films (painted in blue); (**b**) reconstructed conductivity images with injected current frequencies 50 kHz, 70 kHz, 80 kHz, 100 kHz, 200 kHz and 250 kHz. The injection currents at all frequencies up to 70 kHz detoured around the films, and therefore, the bars disturbed by films are not visible in the reconstructed admittivity images at all frequencies up to 70 kHz.

We perform phantom experiments using a 32-channel mfEIT system (EIT-Pioneer Set) made by Swisstom (Swisstom AG, Landquart, Switzerland). The programmable injection current frequency of the Swisstom EIT-Pioneer Set is between 50 kHz and 250 kHz. In Figure 9a, we use a cylindrical phantom (360 mm diameter) with 32 equally-spaced electrodes. The phantom is filled with thick and white porridge with a certain concentration of NaCl. Inside the phantom, we placed steel bars with a diameter of 30 mm and very thin plastic films (1 *μ*m thickness). The thin plastic films are used for modeling insulating cracks; they block the injected currents at low frequencies, while high-frequency currents penetrate them. We inject a current of 1 mA at the six different frequencies of 50 kHz, 70 kHz, 100 kHz, 200 kHz and 250 kHz. (This device has a maximum frequency of 250 kHz.) Collecting the current-voltage data using the 32-channel mfEIT system, we produce mfEIT images at each frequency.

Figure 9b shows the reconstructed images at six different frequencies. The EIT images up to 50 kHz are very similar. At the frequencies up to 70 kHz, currents cannot penetrate the insulating films, so that the current-voltage data are mainly influenced by insulating films. Thus, only insulating films are visible, while the bars are invisible in the reconstructed images. At the frequency of 80 kHz, the reinforcing bars start to be visible. At the frequency above 200 kHz, thin plastic films are invisible. We found that these phantom experiments perfectly match with our numerical simulations.

## 4. Conclusions

This paper provides the advantages of spectroscopic EIT imaging to maximize the information of cracks and reinforcing bars. We get useful insights with the aid of the parameter *β_k_*(*ω*) or *β_δ_*(*ω*) described in this paper. We derived the frequency dependency of the current-voltage data with respect to cracks and reinforcing bars. Phantom experiments match with the numerical simulations. Since the thin insulating cracks appear as capacitors to produce the reactance term, it is desirable to use current injections and to measure voltages at a wide range of frequencies below 10 MHz. The currently available multi-frequency EIT systems (Swisstom EIT-Pioneer Set and KHU Mark 2.5) are able to maintain a signal-to-noise ratio (SNR) of 80 dB between 100 Hz and 250 kHz [23]. On the other hand, the mean SNR in our EIT system decreases significantly beyond 500 kHz. Since measurement at a frequency higher than 500 kHz can be valuable in spectroscopic admittivity imaging, it would be desirable to improve the performance of EIT measurements at high frequencies. We plan to use magnetic induction tomography [24], which is capable of providing the admittivity images in the frequency range of 1 to 10 MHz.

In practical situations, the reliability of the assessment of concrete structures may be affected by various factors, such as the concentration of chlorides in the pore solution and measurement uncertainty due to the contact impedance between the electrodes and the concrete surfaces [18,25]. These factors should be thoroughly investigated in future research studies. Although the proposed method cannot provide high-quality image reconstruction due to the ill-posedness nature of the EIT method, the proposed spectroscopic EIT imaging at multiple frequencies has potential to provide more qualitative information for the assessment of concrete structures.

## Figures and Tables

**Table 1:** Admittivity distribution in each subdomain (ϵ_0_ = 8.85 × 10^−12^ F/m).

**Subdomain**	**Admittivity Distribution**
*D*_1_ *D*_2_	10^5^ + *iω* × 10^6^ × *ϵ*_0_
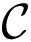 _1_, 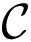 _2_, 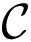 _3_, 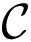 _4_	10^−6^ + *iω* × 10^2^ × *ϵ*_0_
otherwise	1 + *iω* × 10^4^ × *ϵ*_0_

## Appendix

In this Appendix, we briefly summarize the EIT reconstruction method. When we inject a sinusoidal current of I mA with a frequency of *ω*/2*π* between adjacent pair _*j*_, _*j*−1_, as shown in Figure 1, the resulting potential $u_{j}^{\omega }$ is dictated by the following complete model [21,26]:

(A1) $$\{\begin{array}{c} \nabla \cdot \left(\gamma^{\omega }\nabla u_{j}^{\omega }\right)=\;0\;\text{in}\;\Omega ,\\ \left(u_{j}^{\omega }+z_{k}\gamma^{\omega }\frac{\partial u_{j}^{\omega }}{\partial \nu }\right)=U_{j,k}^{\omega }\;\text{on}\;E_{k},k=1,\cdots ,E,\\ \gamma^{\omega }\frac{\partial u_{j}^{\omega }}{\partial \nu }=0\;\text{on}\;\partial \Omega \backslash \cup_{k=1}^{E}E_{k},\\ \int_{E_{k}}\gamma^{\omega }\frac{\partial u_{j}^{\omega }}{\partial \nu }ds=0\;\text{if}\;k\in \left\{1,\cdots ,E\right\}\backslash \left\{j,j-1\right\},\\ \int_{E_{j}}\gamma^{\omega }\frac{\partial u_{j}^{\omega }}{\partial \nu }ds=I=-\int_{E_{j-1}}\gamma^{\omega }\frac{\partial u_{j}^{\omega }}{\partial \nu }ds, \end{array}$$

where *z_k_* is the contact impedance of the *k*-th electrode *_k_* and $U_{\omega }^{j,k}$ is the voltage on the electrode *_k_*.

We sequentially inject currents using all pairs of the adjacent electrodes to collect the voltage dataset **F**_*ω*_1__, ⋯, **F***_ω_K__* at multiple frequencies *ω*_1_, ⋯, *ω_k_*, where:

(A2) $${\text{F}}_{\omega }={\left[V_{1,1}^{\omega },\cdots ,V_{1,E}^{\omega },\cdots ,\cdots V_{E,1}^{\omega },\cdots V_{E,E}^{\omega }\right]}^{T}$$

and $V_{j,k}^{\omega }=U_{j,k}^{\omega }-U_{j,k-1}^{\omega }$. Discretizing the imaging domain Ω into *Q* elements as $\Omega =\cup_{q=1}^{Q}\Omega_{q}$, we can formulate a sensitivity matrix  as:

$$\left(pq\right)-\text{element of}\;S=\int_{\Omega_{q}}\nabla \upsilon_{j}\cdot \nabla \upsilon_{k}d\text{r}\;\text{and}\;p=\left(k-1\right)\times E+j$$

where *ν_j_* is the solution of Equation (A1) with *γ^ω^* being replaced by one and **r** = (*x, y*).

The most widely-used EIT reconstruction method is one-step Gauss–Newton reconstruction with Tikhonov regularization:

$$\gamma^{\omega }=\arg\underset{\delta \gamma }{\min}{\left\|S\gamma^{\omega }-{\text{F}}_{\omega }\right\|}^{2}+\lambda {\left\|\gamma^{\omega }\right\|}^{2}$$

where λ is the regularization parameter. In practice, we use difference EIT imaging through the process of subtracting background components [21,27]. In order to incorporate prior information, statistical inversion techniques, like the Bayesian method, can be used in the EIT problem [28].

## References

[1] Gerard C., Feldmann P.E. (2008). Non-destructive testing of reinforced concrete. Struct. Test..

[2] Colla C., McCann D., Das P., Forde M.C., Binda L., Modena C. (1995). Investigation of a stone masonry bridge using electromagnetics. Joint International Workshop on Evaluation and Strengthening of Existing Masonry Structures.

[3] Davis J.L., Annan A.P. (1989). Ground penetrating radar for high-resolution mapping of soil and rock stratigraphy. Geophys. Prospect..

[4] Diamond G.G., Hutchins D.A., Gan T.H., Purnell P., Leong K.K. (2006). Single-sided capacitive imaging for NDT. Insight.

[5] International Atomic Energy Agency (2002). Guidebook on Non-Destructive Testing of Concrete Structures.

[6] Kim S., Seo J.K., Ha T. (2009). A nondestructive evaluation method for concrete voids: Frequency differential electrical impedance scanning. SIAM J. Appl. Math..

[7] McCann D.M., Forde M.C. (2001). Review of NDT methods in the assessment of concrete and masonry structures. NDT&E Int..

[8] Niemuth M. (2004). Using impedance spectroscopy to detect flaws in concrete. Master's Thesis.

[9] Pour-Ghaz M., Weiss J. (2011). Detecting the time and location of cracks using electrically conductive surfaces. Cem. Concr. Compos..

[10] Pour-Ghaz M., Weiss J. (2011). Application of frequency selective circuits for crack detection in concrete elements. J. ASTM. Int..

[11] Pour-Ghaz M., Niemuth M., Weiss J., Glisic B. (2012). Use of electrical impedance spectroscopy and conductive surface films to detect cracking and damage in cement based materials. Structural Health Monitoring Technologies—Part I..

[12] Pour-Ghaz M., Barrett T., Ley T., Materer N., Apblett A., Weiss J. (2014). Wireless crack detection in concrete elements using conductive surface sensors and radio frequency identification technology. J. Mater. Civ. Eng..

[13] Sansalone M., Carino N.J. (1988). Impact-echo method: Detecting honeycombing, the depth of surface-opening cracks, and ungrouted ducts. Concr. Int. Des. Cons..

[14] Sansalone M., Carino N.J. (1986). Impact-echo: A method for flaw detection in concrete using transient stress waves.

[15] Soleimani M., Stewart V., Budd C. (2011). Crack detection in dielectric objects using electrical capacitance tomography imaging. Insight Non-Destruct. Test. Cond. Monit..

[16] Yin X., Hutchins D.A., Diamond G.G., Purnell P. (2010). Non-destructive evaluation of concrete using a capacitive imaging technique: preliminary modelling and experiments. Cem. Concr. Res..

[17] Hou T.C., Lynch J.P. Tomographic imaging of crack damage in cementitious structural components.

[18] Karhunen K., Seppänen A., Lehikoinen A., Blunt J., Kaipio J.P., Monteiro P.J.M. (2010). Electrical resistance tomography for assessment of cracks in concrete. ACI Mat. J..

[19] Karhunen K., Seppänen A., Lehikoinen A., Kaipio J.P., Monteiro P.J.M., Alexander M.G., Beushausen H.-D., Dehn F., Moyo P. (2009). Locating reinforcing bars in concrete with electrical resistance tomography. Concrete Repair, Rehabilitation and Retrofitting II.

[20] Karhunen K., Seppänen A., Lehikoinen A., Monteiro P.J.M., Kaipio J.P. (2010). Electrical resistance tomography imaging of concrete. Cem. Concr. Res..

[21] Seo J.K., Woo E.J. (2012). Nonlinear Inverse Problems in Imaging.

[22] Holder D. (2005). Electrical Impedance Tomography: Methods, History and Applications.

[23] Oh T.I., Woo E.J., Holder D. (2007). Multi-frequency EIT system with radially symmetric architecture: KHU Mark1. Physiol. Meas..

[24] Griffiths H. (2001). Magnetic induction tomography. Meas. Sci. Technol..

[25] Enevoldsen J.N., Hansson C.M. (1994). The influence of internal relative humidity on the rate of corrosion of steel embedded in concrete and mortar. Cem. Concr. Res..

[26] Somersalo E., Cheney M., Isaacson D. (1992). Existence and uniqueness for electrode models for electric current computed tomography. SIAM J. Appl. Math..

[27] Oh T.I., Koo H., Lee K.H., Kim S.M., Lee J., Kim S.W., Seo J.K., Woo E.J. (2008). Validation of a multi-frequency electrical impedance tomography (mfEIT) system KHU Mark1: Impedance spectroscopy and time-difference imaging. Physiol. Meas..

[28] Kaipio J.P., Kolehmainen V., Somersalo E., Vauhkonen M. (2000). Statistical inversion and Monte Carlo sampling methods in electrical impedance tomography. Inverse Probl..

